# Supplementary material for: Beyond diagnostic connectivity: Leveraging digital health technology for the real-time collection and provision of high-quality actionable data on infectious diseases in Uganda
Source: PLOS Digit Health. 2024 Aug 23;3(8):e0000566. doi: 10.1371/journal.pdig.0000566 (PMC11343378; doi:10.1371/journal.pdig.0000566)

PLOS Digital Health

Beyond diagnostic connectivity: leveraging digital health technology for the real-time collection and provision of high-quality actionable data on infectious diseases in Uganda

--Manuscript Draft--

**Manuscript Number:**	PDIG-D-23-00344
**Article Type:**	Research Article
**Full Title:**	Beyond diagnostic connectivity: leveraging digital health technology for the real-time collection and provision of high-quality actionable data on infectious diseases in Uganda
**Short Title:**	Beyond diagnostic connectivity: leveraging digital health technology for disease control
**Corresponding Author:**	Dennis Mujuni, MSc.<br>Makerere University CHS: Makerere University College of Health Sciences<br>Kampala, Non-US/Non-Canadian UGANDA
**Order of Authors:**	Dennis Mujuni, MSc.
	Julius Tumwine
	Kenneth Musisi, MSc.
	Dorothy Nabulobi, MSc.
	Maha Reda Farhat, PhD
	Nyombi Abdunoor
	Arnold Kennedy Tumuhairwe, BSc.
	Marvin Derrick Mugisa, BSc.
	Denis Oola, MSc.
	Edward Otim
	Fred Semitala
	Raymond Byaruhanga, MSc.
	Stavia Turyahabwe, MSc.
	Moses Joloba
**Keywords:**	Diagnostic data connectivity; Disease surveillance; Digital health technology
**Abstract:**	Automated data transmission from diagnostic instrument networks to a central database at the Ministries of Health has the potential of providing real-time quality data not only on diagnostic instrument performance, but also continuous disease surveillance and patient care. We aimed at sharing how a locally developed novel diagnostic connectivity solution channels actionable data from diagnostic instruments to the Uganda national dashboards between May 2022 and May 2023 for disease control in Uganda. The diagnostic connectivity solution was successfully configured on a selected network of multiplexing diagnostic instruments at 260 sites in Uganda, providing a layered access of data. Of these, 912,144 test results were automatically collected from 269 “GeneXpert” machines, 5597 test results from 39 “Truenat” and >12,000 were from 3 digital x-ray devices to different stakeholder levels to ensure optimal use of data for their intended purpose. The government and relevant stakeholders are empowered with usable and actionable data from the diagnostic instruments. The successful implementation of the diagnostic connectivity solution depended on some key operational strategies namely; sustained internet connectivity and short message services, stakeholder engagement, a strong in-country laboratory coordination network, human resource capacity building, establishing a network for the diagnostic instruments, and integration with existing health data collection tools. Poor bandwidth at some locations was a major hindrance for the successful implementation of the connectivity solution.

Powered by Editorial Manager® and ProduXion Manager® from Aries Systems Corporation

	Maintaining stakeholder engagement at the clinical level is key for sustaining diagnostic data connectivity. The locally developed diagnostic connectivity solution as a digital health technology offers the chance to collect high-quality data on a number of parameters for disease control, thereby strengthening the quality of data from the networked diagnostic sites to relevant stakeholders.
**Additional Information:**	
**Question**	**Response**
**Government Employee**<br><br>Are you or any of the contributing authors an employee of the United States government?<br><br>Manuscripts authored by one or more US Government employees are not copyrighted, but are licensed under a CC0 Public Domain Dedication, which allows unlimited distribution and reuse of the article for any lawful purpose. This is a legal requirement for US Government employees.<br><br>This will be typeset if the manuscript is accepted for publication.	No - No authors are employees of the U.S. government.
**Financial Disclosure**<br><br>Enter a financial disclosure statement that describes the sources of funding for the work included in this submission and the role the funder(s) played. This includes grants and any commercial funding of the work or authors.<br><br>This statement will be typeset if the manuscript is accepted for publication.<br><br>Review the submission guidelines and the instructions link below for detailed requirements and guidance.	This implementation of the digital health technology was supported by a grant from The United States Agency for International Development (USAID) through the Stop TB Partnership and the Introducing New Tools Project (iNTP) Grant Number STBP/NT/GSA/2022-02. The iNTP project generally aims to strengthen TB care in high-burden nations by introducing a suite of cutting-edge innovations in diagnostics, therapies, and digital health technologies. The funder had no specific role in the conceptualization, design, data collection, analysis, decision to publish, or preparation of the manuscript.
**Competing Interests**<br><br>On behalf of all authors, disclose any competing interests that could be perceived to bias this work.	The authors declare that they have no competing interests.

Powered by Editorial Manager® and ProduXion Manager® from Aries Systems Corporation

This statement will be typeset if the manuscript is accepted for publication.<br><br>Review the instructions link below and PLOS Digital Health's competing interests policy to determine what information must be disclosed at submission.	
**Data Availability**<br><br>Provide a **Data Availability Statement** in the box below. This statement should detail where the data used in this submission can be accessed. This statement will be typeset if the manuscript is accepted for publication.<br><br>Before publication, authors are required to make all data underlying their findings fully available, without restriction. Review our PLOS Data Policy page for detailed information on this policy. Instructions for writing your Data Availability statement can be accessed via the Instructions link below.	The datasets used and/or analyzed during the current study are owned by the government of Uganda and are therefore only available from the corresponding author on reasonable request.

Manuscript

Click here to access/download;Manuscript;Beyond diagnostic connectivity.docx

Beyond diagnostic connectivity: leveraging digital health technology for the real-time collection and provision of high-quality actionable data on infectious diseases in Uganda

Dennis Mujuni[1*], Julius Tumwine[2], Kenneth Musisi[2], Dorothy Nabulobi[2], Maha Reda Farhat[3], Nyombi Abdunoor[2,5], Arnold Kennedy Tumuhairwe[4], Marvin Derrick Mugisa[2], Denis Oola[2], Edward Otim[5], Fred Semitala[5], Raymond Byaruhanga[6], Stavia Turyahabwe[6], Moses Joloba[2],

[1]Makerere University, College of Health Sciences, Kampala, Uganda.

[2]Uganda National TB Reference Laboratory, World Health Organisation Supranational Reference Laboratory, Kampala, Uganda.

[3]Department of Medical Informatics, Harvard Medical School, Harvard University, Boston, Massachusetts, United States.

[4]Makerere University, School of Social Sciences, Kampala, Uganda.

[5]Makerere University Joint AIDS Program, Kampala, Uganda.

[6]National Tuberculosis and Leprosy Control Program, Ministry of Health, Kampala, Uganda.

Corresponding author

Correspondence to Dennis Mujuni dennismujuni.n@gmail.com

Abstract

Automated data transmission from diagnostic instrument networks to a central database at the Ministries of Health has the potential of providing real-time quality data not only on diagnostic instrument performance, but also continuous disease surveillance and patient care. We aimed at sharing how a locally developed novel diagnostic connectivity solution channels actionable data

from diagnostic instruments to the Uganda national dashboards between May 2022 and May 2023 for disease control in Uganda. The diagnostic connectivity solution was successfully configured on a selected network of multiplexing diagnostic instruments at 260 sites in Uganda, providing a layered access of data. Of these, 912,144 test results were automatically collected from 269 “GeneXpert” machines, 5597 test results from 39 “Truenat” and >12,000 were from 3 digital x-ray devices to different stakeholder levels to ensure optimal use of data for their intended purpose. The government and relevant stakeholders are empowered with usable and actionable data from the diagnostic instruments. The successful implementation of the diagnostic connectivity solution depended on some key operational strategies namely; sustained internet connectivity and short message services, stakeholder engagement, a strong in-country laboratory coordination network, human resource capacity building, establishing a network for the diagnostic instruments, and integration with existing health data collection tools. Poor bandwidth at some locations was a major hindrance for the successful implementation of the connectivity solution.

Maintaining stakeholder engagement at the clinical level is key for sustaining diagnostic data connectivity. The locally developed diagnostic connectivity solution as a digital health technology offers the chance to collect high-quality data on a number of parameters for disease control, thereby strengthening the quality of data from the networked diagnostic sites to relevant stakeholders.

Keywords: Diagnostic data connectivity, Disease surveillance, Digital health technology

Author Summary

Uganda, like many Lower and Middle-Income Countries (LMICs), is investing in World Health Organization Recommended Diagnostic (WRD) tools to diagnose communicable diseases. Despite our efforts to improve case detection and treatment initiation, we face a significant challenge when

it comes to data transmission, mainly relying on paper-based systems. Emphasis on the importance of diagnostic connectivity is crucial to make the most of the digital data generated by these diagnostic tools.

In our research, we explore innovative approaches to enhance infectious disease control in Uganda through the adoption of digital health technology. While the digital health platform has shown great potential beyond just diagnostic connectivity for results transmission, it does come with its set of challenges. This platform requires internet connectivity for the automated data upload and relies on human intervention to verify patient and clinician contact details for effective notifications.

Our paper aims to raise awareness of the possibilities that digital health technology offers for laboratories and disease control programs in resource-constrained areas. It serves as a diagnostic data connectivity tool that goes beyond mere diagnostic connectivity and holds the promise of gathering high-quality data on disease diagnosis, instrument performance, continuous disease surveillance, and even patient care.

Introduction

In Lower and Middle Income Countries (LMICs), infectious diseases like tuberculosis (TB) and human immunodeficiency virus (HIV) have significantly influenced the design and planning of diagnostic networks with heavy reliance on manual procedures and expert consensus, and only a limited use of data analytics [1]. The recent years have also seen an increased uptake of diagnostics for infectious diseases, including TB and HIV, human papilloma virus (HPV) and corona virus (CoV) at points of care among others, although discrepancies in the number of cases reported to national authorities have been cited [2]. For instance, an estimated 3.6 million persons with

tuberculosis were either not detected or not reported to local authorities, and hence went unnoticed by formal health systems in 2017 alone [3]. Many of these unidentified people with tuberculosis do not receive the care they require, putting them at risk of developing serious and sometimes deadly infections as well as being a possible source of transmission to others around them [3,4].

We recently demonstrated that in settings with limited resources, the National TB Control Programs (NTPs) may benefit from real-time surveillance of evolving tuberculosis drug resistance by using routinely generated laboratory data from current molecular diagnostic methods [5]. It is essential that such data from diagnostic instruments be networked for programmatic decision-making, later reviews to address important research issues on areas of disease control [6] including complementary testing for improved TB case detection, data gathering, and even linkages to care for TB disease. This may be achieved through the implementation of digital health technology.

Digital health has seen significant growth and development in the recent years [7], with the emergence of new technologies that aim to improve healthcare delivery and patient outcomes [8,9]. One such technology is the locally developed novel diagnostic connectivity solution, which automates data synchronization from diagnostic instruments like "GeneXpert", digital X-ray and "Truenat" machines. Traditionally, diagnostic instruments generate data that is stored in disparate systems and may require manual entry into electronic management records (EMRs) [10], which can be time-consuming and error-prone. With the development of this digital health solution, data synchronization is automated, enabling healthcare providers to streamline their workflows.

The solution works by integrating with the various diagnostic and screening instruments and automatically extracting the relevant data from the diagnosis of communicable diseases like TB, HIV, HPV, and CoV, which is then synchronized to a national server for displays and visualization on a dashboard in real-time. This not only reduces the burden on healthcare providers but also

improves the accuracy and completeness of patient records, which can lead to better diagnosis and treatment decisions.

The benefits of digital health technology are numerous, including improved efficiency, increased data accuracy, and enhanced patient outcomes [11]. Furthermore, it has the potential to revolutionize healthcare delivery by enabling healthcare providers to focus on what they do best – providing high-quality patient care [12]. In this paper, we present our experience on how the locally developed diagnostic connectivity solution may be used as a digital health technology that goes beyond connectivity to make the data from the WHO recommended diagnostic platforms such as “GeneXpert”, “Truenat” and digital chest X-ray (CXR) systems actionable and useful for the effective control of communicable diseases.

Results

Diagnostic Network Connectivity

The locally developed diagnostic connectivity solution has been successfully configured on 269 “GeneXpert” machines at 260 “GeneXpert” testing sites, 39 “Truenat” machines and digital chest X-ray machines countrywide. These diagnostic sites are automatically classified as "Active" if their machine sent a report within the previous 24 hours, "Dormant" if no report was sent within the previous 2 to 4 days, and "Inactive" if no test data was sent within the past 4 days or longer, Figure 1.

Figure 1: Reporting trends for “GeneXpert” machines across the selected quarters

By the end of May 2023, the platform had gathered a total of 912,144 test results from “GeneXpert” machines, 5597 from “Truenat” machines, and >12,000 results from the Chest X-Rays. Of the 912,144 test results from “GeneXpert”, 845,938 were attributed to Tuberculosis, 31,117 to HIV, 8065 to COVID-19 and 24,554 to HPV.

Data accessibility, usability and ownership

The novel diagnostic connectivity solution empowers the government and relevant stakeholders with usable and actionable data from the diagnostic instruments. The platform restricts unauthorized access while providing a layered access of data at different stakeholder levels to ensure optimal use of data for its intended purpose. Aggregated (raw) data from the diagnostic connectivity solution provides an opportunity for the automated synchronization with the Ministry of Health District Health Information Software 2 (DHIS2) platform. Key human resources are able to monitor and act on the data made available to them from the platform to include test results, drug-resistant TB cases, equipment management, inventory, errors, and/or other available data as the platform is further developed.

For instance, "GeneXpert" summary indicators for tuberculosis indicated that 832,104 tests were done, consisting of 41,978 MTB positive, 1797 Rifampicin resistant, 736,464 MTB Negative, 9417 Rifampicin Indeterminate, 42,448 Errors as demonstrated in Figure *2* below.

Figure 2: GeneXpert TB indicators of data from January 2020 to May 2023

For Truenat, the summary indicators for tuberculosis indicated that of the 5389 total tests done, 263 were MTB Positive, 4459 MTB Negative, and 667 Errors, Figure *3*.

Figure 3: Truenat TB data indicators from the pilot phase between August 2022 and May 2023

Addressing Diagnostic Utilization Data

The integration of the locally developed novel diagnostic connectivity solution has allowed implementing partners and all testing sites to monitor the minimum number of examinations per unit per day at a given time on their designated dashboards from the captured data. Sites have been empowered with data visualization that enables them keep track of their utilization based on

consumption rate of stock while also accessing summary reports on positivity rates, critical results and workload among others. The diagnostic machine utilization trends can be visualized at different stakeholder levels for action, Figure *4*.

Figure 4: GeneXpert TB utilization trends based on quarterly data from reporting sites between January 2023 and March 2023

Digital epidemiology

The traditional model for public health disease surveillance has always been through the public, public health practitioners, laboratory data, ministries of health, world bodies such as the United Nations, World Health Organization, among others [13]. Digital epidemiology presents the opportunity to work it the other way round, with information being fed to these different parties at the click of a button, as in Figure *5* below.

Figure 5: Digital epidemiology showing near real-time disease surveillance based on weekly data from reporting sites within a period of interest

A reward system

The diagnostic connectivity solution has a performance reward system that recognizes individual efforts in terms of machine utilization, connectivity among other parameters per site. Individuals are able to compare themselves against colleagues at other sites using the ranking system for motivation. The reward system awards points based on daily utilization of the machine, low error rates, among others, and then grades the performance with Gold, Silver and Bronze status depending on the points scored.

Equipment performance monitoring and reporting

With the locally developed diagnostic connectivity solution, we have introduced a user-friendly dashboard feature for alerts that would facilitate adherence to equipment service and maintenance schedules through reminders on service and maintenance, while also highlighting machine performance problems. Data on error alerts, all replaced modules, the non-functional modules, which particular modules are associated with errors, etcetera are also well represented. Targeted interventions can then be directed based on the presented error data as in the case of "GeneXpert" machines as shown in Figure *6* below.

Figure 6: Trends of GeneXpert error rates based on data from reporting sites within a selected time frame

Linkage to care

The platform strengthens patient linkage to care by facilitating for the electronic tracking of positively diagnosed TB patients from "GeneXpert", "Truenat", and the "CAD4TB™" Chest X-Ray system. By the end of May 2023, more than 35,000 notifications had been sent to clinicians and/or patients about the result and readiness of their "GeneXpert" TB test result at the facility respectively.

Integrating the diagnostic connectivity solution with the existing health data management tools

At the time of this paper, initiatives were underway to ensure the integration with the Electronic Case-Based Surveillance System (eCBSS) and District Health Information Software 2 tools as our approach to increase TB case notification and allow for data synchronization to enhance the quality of data available at national level.

Sample accountability across the testing cascade

With our digital health technology, we have laid a foundation to usher in for the first time, the possibility for the accountability of patients' samples through the dashboard after they are shipped from referring facilities for culture and drug susceptibility testing (DST) to the NTRL. This in turn facilitates the tracking of samples as they are transported by hub riders who use motorcycles in the popular hub system of Uganda [14,15].

Discussion

Diagnostic network connectivity

Automated data transmission can be made possible by networking point of care (POC) devices to a central database at the Ministries of Health, thereby providing the ability for real-time data on diagnostic instrument performance [16]. From our early implementation experience, some sites do not report on a daily basis and report within a range of 4 days. Recent literature has cited that infrastructural gaps, limited internet connectivity, and absence of clear policies and legislation weaken reporting [17]. The assumption that reporting can be resolved by ensuring internet connectivity all time through is not entirely true. We have observed that a reward system that recognizes personnel efforts at testing facilities may be a factor in attempt to complete the diagnostic reporting puzzle. The World Health Organization guidelines on digital health interventions [18] underlined a caution stressing that the implementation of a digital health intervention requires a change in current practice or behavior and therefore strong government leaders to promote that change [19].

As an extra effort measure, in the case of Uganda, a TB regional coordinator is assigned to each of the 15 regions and this person may prove to be helpful in following up with sites that do not report within 2 days even when internet bundles have been procured. This has demonstrated the potential of increasing real-time data collection from these sites.

Recent research has shown the potential advancements that include aspects such as Internet of medical things, where medical professionals can obtain real-time data on patients' health conditions, which can help in the early detection, improve patient outcomes and reduce healthcare costs of infectious diseases [20]. We have shown that it is possible to pick multiple data from various disease assays such as HIV viral load, COVID-19 and HPV from the machines at various locations simultaneously without major data interoperability challenges. Other stakeholders can mobilize resources and leverage the connectivity solution in place, especially with the already established infrastructure in place to support integrated data collection on other infectious diseases.

“Truenat” is being piloted to increase molecular testing capacity for rifampicin and isoniazid and runs off grid on a powered by a battery [21]. The application of chest X-ray as a quick imaging method for risk stratification and testing for lung anomalies that indicate asymptomatic active thoracic cavity disease is limited. The method has been recommended for use in patients living with HIV/AIDS, as well as those at high risk of HIV infection, after first negative bacteriological tests to detect tuberculosis [22]. The integration with both “Truenat” and the “CAD4TB™” Chest X-Ray system has allowed for a unidirectional flow of data without duplication that would result into over reporting and data inaccuracies. This step will ably continue to facilitate the linkage of all bacteriologically confirmed TB cases for further testing on “GeneXpert” and/or “Truenat” diagnostic systems and subsequent anti-TB treatment initiation. The digital health technology dashboards has been built to keep track of the percentage linkage of the CXR confirmed cases for further testing, treatment initiation and surveillance.

Utilization of data

The need for intervention whenever sites are underutilizing the deployed TB diagnostic instruments makes it imperative to intensify approaches to actively monitor and enable relevant

program officers intervene in a timely manner. The Uganda NTP currently defines the desirable utilization at a “GeneXpert” facility as 3 tests per day for each available module, which translates into 12 tests per day for 4 module “GeneXpert” machines. The same definition categorically stands for “Truenat” in terms of the turnaround time in context to the number of working hours per day. The maximum testing capacity of the “CAD4TB™” CXR system is 300 per unit per day and future efforts after the pilot phase may have to define the desirable utilization cap. The locally developed novel diagnostic connectivity solution has demonstrated its potential usefulness, in assessing the performance of the TB instrument networks by availing various data for the country to realize their full potential. This approach has the potential to strengthen data utility at different stakeholder levels [23,24]. In terms of sustained service delivery, our experience has shown that personnel can closely monitor their stock since anecdotal evidence from previous support supervisions have consistently reported stock outs for a pitfall, as patients suffer delayed diagnosis or unfortunately miss out on it as a result of stock-outs.

The map features from the platform also support disease surveillance mechanisms in real time, as program officers and other key stakeholders get to see hotspots for TB in its different forms as a complement to the eCBSS and DHIS2 tools. It has been highlighted that the adoption of digital health technology for disease surveillance purposes creates complex challenges, which necessitates the development of tailor made solutions for sub-Saharan Africa [25]. However, recent updates highlight the need to update infectious disease datasets to account for the fraction that is not collected by the surveillance system because infectious diseases are particularly known to be vulnerable to inadequate representation by raw surveillance data [26]. Uganda currently uses robust electronic health data management systems, such as the District Health Information Software 2 and the Electronic Case-Based Surveillance System. The DHIS2 is currently used to

aggregate statistical data collection, validation, analysis, management, and presentation. This data analytics and management platform is completely web-based and boasts great visualization features and the ability to create analysis from live data in seconds [27]. Case-based surveillance, on the other hand, is the technique of collecting patient-level data for a set of key or reportable sentinel events in order to measure and monitor the incidence, course, and outcome of a disease [28].

Building technical capacity

Human resource capacity is a key element for usability of the connectivity solution at the different testing sites since inadequate HR capacity has been a major hindrance in efforts to monitor and act upon the data. For instance, the trained human resource was taken through the aspect of entering stock upon receipt from the national stores in order to facilitate the accurate computation rate of stock consumption which in turn allows for proper forecasted logistics and accountability. They were also empowered with knowledge to do with troubleshooting errors and their significance on machine functionality.

Prior to the introduction of “Truenat” and the “CAD4TB™” Chest X-Ray system, Uganda among other countries encountered persistent challenges in adhering to equipment service and maintenance schedules for the “GeneXpert” platform. With the features to do with equipment management, the locally developed diagnostic connectivity solution may potentially make it possible to increase machine performance since it has a functionality that allows the sharing of instrument network performance data with manufacturers and/or partners to be able to troubleshoot and/or establish better reporting systems of service for the machines. These data computations facilitated by the diagnostic connectivity solution may enable diligence in adherence to equipment service and maintenance schedules from the authorized service providers with attrition to

functionalities that include service and maintenance reminders, equipment downtime recording, acceptable and unacceptable error alerts on dashboard.

Lastly, linkage to care is a very critical step in the successful treatment and management of infectious diseases [29,30]. In this section of our paper, we discuss how the locally developed novel diagnostic connectivity solution can be useful for linking patients suffering from Tuberculosis in all its forms (susceptible or resistant) for successful treatment and management. TB linkage to care can be defined as the completion of a clinical visit after TB diagnosis is done [31] and remains a necessary precursor to initiation of anti-TB treatment. Successful linkage to care may therefore be primarily defined as registration for anti-TB therapy within 28 days after TB diagnosis [32]. The WHO widely recommends timely linkage to care to ensure that patients access appropriate treatment in a timely manner. Often times than not, the delay for initiation or change of anti-TB therapy is because the patient sample delayed to reach the NTRL in time, the sample quality was not fulfilling minimum sample reception requirements, or was never sent at all. In Uganda, the results between referring to testing sites ultimately contribute to how soon rifampicin resistant tuberculosis (RR-TB) samples are referred to the NTRL for further DST [33]. With the implemented digital health technology, functionalities such as SMS prompts to patients in communities, and to clinicians at facility level, as well as the MDR sample tracking component at the NTRL level present an opportunity to make it possible for the timely linkage of patients for the intended care and management. We acknowledge the fact that more sensitization is needed for clinicians and patients to appreciate this notification feature. Evaluating the utility of this intervention in its entirety at different stakeholder levels may be helpful, most especially in improving TB clinical care in Uganda and other similar settings.

Limitations

The project encountered a few technical challenges, most especially differences in the software versions on "GeneXpert" machines, where we noted that versions 4.7b, 5.1 and 5.3 presented with system incompatibility. This was addressed by ensuring that the respective machines were updated to the later versions that were compatible with the digital health technology prior to system configurations.

The platform also requires internet connectivity for the automated upload of data, and relies on human intervention for the verification of patient and clinician contact details for effective notifications. This was one of the key challenges associated with the application of digital health for universal health coverage in Africa by a recent review [34]. The efficient implementation of the connectivity solution is greatly hampered by inadequate internet bandwidth at some places, especially health facilities at islands. Retrieving previous information was also problematic, as not all tests done prior to configurations were automatically uploaded to the designated server, thereby affecting reporting. Furthermore, monitoring the machine's functionality based on the usage presents a limitation, as the upload of results is contingent upon conducting tests at the testing site.

Conclusions

The experiences from the locally developed novel diagnostic data connectivity platform are generalizable to other settings as it exceeds mere diagnostic connectivity and promises the possibility to gather high-quality data on diagnosis, instrument performance, continuous disease surveillance, as well as improving patient care. Sustained diagnostic data connectivity may minimize transcriptions of data from paper-based registers into the District Health Information Software (DHIS) platform of the Ministry of Health for other disease areas, thereby strengthening the quality of data from the networked diagnostic sites.

Materials and Methods

Study aim, design and setting

The study was aimed at improving connectivity and data sharing among TB diagnostic instrument networks. The study employed a pre-post intervention design where a team conducted an exercise to establish and implement a connectivity solution for the TB diagnostic instrument networks in Uganda between May 2022 and May 2023. This digital health technology was implemented in a series manner across the 15 TB regions.

Ethics approval and consent to participate

All methods were carried out ethically, according to the Uganda National Guidelines for Research involving Humans as Research Participants-(July-2014.page 28) given that this study involved the use of human data from patient samples routinely collected for patient care.

The Uganda Ministry of Health NTRL Clinicians Handbook (Version 4.0, February-2019, Page 7 of 20) states that a patient who voluntarily moves to the laboratory to provide a sample has already consented and that informed consent does not need to be obtained from the patient whose samples are going to be used in their care and management. This also includes subjects with legal guardians. As a result, according to the aforementioned national regulations, the need for informed consent was deemed unnecessary and was no longer required.

Institutional ethical approval in this accordance was therefore obtained from the National Disease Control Department under the National Tuberculosis and Leprosy Program of the Ministry of Health, Uganda.

Methodology

A narrative and description of the activities that were implemented the diagnostic connectivity solution for the TB diagnostic instrument networks in Uganda is broken down below;

Engagement of stakeholders

The activities for implementing a locally developed novel platform as a diagnostic data connectivity solution begun with a hybrid engagement of stakeholders, for both virtual and physical discussions. This group of stakeholders included program officers from the Ministry of Health, National TB Control Program, National TB Reference Laboratory (NTRL), key implementing partners and donors in the sector. This engagement entailed onboarding the stakeholders on the project goals and objectives in order to receive relevant support on ground, and also useful suggestions for the smooth running of project activities.

Setting up a diagnostic data connectivity network in the country

The central focus of this paper is to demonstrate the possibility of setting up a robust network infrastructure through which diagnostic platforms can be interconnected countrywide using digital health technology. We achieved this through the following activities; (i) Setting up a network infrastructure using routers, (ii) Installing the locally developed novel diagnostic connectivity solution on the network of TB diagnostic machines to include “GeneXpert”, “Truenat” and digital chest X-ray machines to save users the need for manual intervention for results to be retrieved from machines. This was done by performing a configuration of the server address into the host communication ports of the diagnostic platform. The American Society for Testing and Materials (ASTM) protocol was selected and host communication enabled after defining the host test codes accordingly. Briefly, the diagnostic platforms were configured via the host communication through the system configuration dialog, where a specific server internet protocol (IP) address and port number were entered. An IP address is a unique numerical label that is connected to a computer network that governs the format of data sent via the internet or local network [35]. The port number on the other hand routes the internet communication or other network message to specific

processes within the diagnostic connectivity platform by using a port number when it reaches a server [36].

We took precautionary steps by considering issues raised in studies conducted in the line of developing priority digital products and making them work not only for the ENDTB Strategy but also sustainable development goals [37,38].

Piloting and enrolling the multi-drug resistant (MDR) management module.

We have integrated the MDR management module seeking to close the growing gap between persons who have been diagnosed with tuberculosis with special interest in DR-TB and those who have begun anti-TB medication. We laid the foundation for DR-TB notification and registration, with capabilities for follow-up and management, which shall follow with availability of resources.

Integrating with existing data collection tools

Uganda's journey to a web-based electronic case-based surveillance system began in 2013 with the launch of the Uganda Global Health Security (GHS) Demo Project by the Federal Government of the United States [28]. The DEFEAT TB project supported the development and roll out of the e-case based surveillance system [39] for the NTP in order to ensure the close monitoring of MDR-TB patients throughout their treatment cycle. However, a gap has been evident in the sending of data from the testing laboratory for registry into the eCBSS. The integration of the locally developed diagnostic connectivity solution with the Electronic Case-Based Surveillance System and DHIS2 is on course, such that aggregated data or raw data from the connectivity solution will automatically be entered in the Ministry of Health (MOH) DHIS2 platform. With the successful configuration of the locally developed diagnostic connectivity solution, all patient laboratory data would automatically be registered in the eCBSS.

By leveraging the automated statistical analysis and data visualization capability, our study benefited from timely and evidence-based decision-making. Healthcare administrators gained actionable insights to allocate resources efficiently, policymakers obtained a comprehensive overview of TB prevalence and response, and diagnostic instrument operators could identify areas for improvement in real time.

This innovative approach not only streamlined the data analysis process but also empowered stakeholders to make informed decisions that positively impacted TB diagnosis and treatment across Uganda. Through the automatic statistical analysis and intuitive data visualizations, our connectivity solution revolutionized the way data-driven decisions are made in the context of TB diagnostics.

Training of users and other relevant human resources

The training of users begun at national level, such that the trained national level persons are able to handle the coordination of key project activities for the support of users across the different laboratories in the country. Subsequently, end-user training was conducted at the regional level across all 15 health regions of the country. This entailed leveraging the expertise of the teams previously trained at the national level to provide technical support for the regional training sessions.

Quality assurance supervision

After the completion of national and regional trainings, designated teams conducted a series of quality assurance supervisions to undertake project monitoring and evaluation (M&E) and provide support to health facilities in addressing challenges. These efforts were implemented to meet the grant award requirements, which call for quarterly project progress reports to simplify intervention and final reporting.

Confidentiality policy efforts and mechanisms

The SMS prompts are sent by the server to the mobile network and receipt is only observed on the patient's handset or that availed as the next of kin/care taker in the event that the patient has no phone at the time of enrolment at the testing facility. The patients receive a notification on the readiness of results from the testing site once the test is completed. We opted to take note of the care taker’s contact details in instances where the patient had no phone number because this was the most significant point of attrition for missed notifications in the study conducted by Babirye *et al.*, in 2019 [40].

Data privacy and ownership

The connectivity solution provides different access rights at different levels of the organization to ensure optimal use of data for the intended purpose at facility level. This is ensured through the login requirements for individual sites, ultimately restricting unauthorized access.

The SMS for the health facility persons enrolled is sent by the server, which is delivered through the mobile network and received on the respective registered handset(s). Stakeholders need to take further steps to ensure that the participating clinicians follow up patients with a call to the patients/caretakers. Considering follow-up calls as a supplementary pathway is justified based on a prior study conducted in Uganda, which confirmed that the health center was able to effectively deliver and receive a majority of “GeneXpert” SMS results [40]. All data generated from the connectivity solution is owned by the government of Uganda.

Statistical analysis

In our study, the implemented diagnostic connectivity solution facilitated a sophisticated and automated data analysis process of the data collected from various TB diagnostic instruments across the 15 regions of Uganda. This analysis encompassed key metrics such as diagnostic

utilization rates for different disease types, testing error frequency, diagnostic logistic utility, and regional variations in the same parameters among others.

The diagnostic connectivity solution further provided real-time data visualization through dynamic and interactive graphs, charts, and dashboards. These visualizations allowed healthcare professionals and stakeholders to grasp trends, patterns, and correlations within the diagnostic network. Additionally, the system generated automated reports, summarizing the statistical findings and highlighting key observations.

Acknowledgements

Administrators at the Uganda National Tuberculosis Reference Laboratory and the Makerere Joint AIDS Program are greatly acknowledged for their overall support of this piece of work. The project employees and members of the health teams at the health facilities in Uganda, that contributed to the generation and implementation of the platform presented in this study are also recognized in a special way for their technical assistance, co-operation, and efforts within the connectivity project.

References

1. Nichols K, Girdwood SJ, Inglis A, Ondoa P, Sy KTL, Benade M, et al. Bringing Data Analytics to the Design of Optimized Diagnostic Networks in Low- and Middle-Income Countries: Process, Terms and Definitions. Diagn Basel Switz. 2020;11: E22. doi:10.3390/diagnostics11010022

2. Land KJ, Boeras DI, Chen X-S, Ramsay AR, Peeling RW. REASSURED diagnostics to inform disease control strategies, strengthen health systems and improve patient outcomes. Nat Microbiol. 2019;4: 46–54. doi:10.1038/s41564-018-0295-3

3. World Health Organisation. Global TB Report 2018. Sep 2018 [cited 3 Feb 2021]. Available: https://apps.who.int/iris/handle/10665/274453

4. Knechel NA. Tuberculosis: Pathophysiology, Clinical Features, and Diagnosis. Crit Care Nurse. 2009;29: 34–43. doi:10.4037/ccn2009968

5. Mujuni D, Kasemire DL, Ibanda I, Kabugo J, Nsawotebba A, Phelan JE, et al. Molecular characterisation of second-line drug resistance among drug resistant tuberculosis patients tested

in Uganda: a two and a half-year's review. BMC Infect Dis. 2022;22: 363. doi:10.1186/s12879-022-07339-w

6. Taaffe J, Croda J, Moultrie H, Silva DS, Rosenthal A, Farhat M. Advancing TB research using digitized programmatic data. Int J Tuberc Lung Dis. 2021;25: 890–895. doi:10.5588/ijtld.21.0325

7. Cuff A. The evolution of digital health and its continuing challenges. BMC Digit Health. 2023;1: 3. doi:10.1186/s44247-022-00004-x

8. Abernethy A, Adams L, Barrett M, Bechtel C, Brennan P, Butte A, et al. The Promise of Digital Health: Then, Now, and the Future. NAM Perspect. 2022;2022: 10.31478/202206e. doi:10.31478/202206e

9. McGinnis JM, Fineberg HV, Dzau VJ. Advancing the Learning Health System. N Engl J Med. 2021;385: 1–5. doi:10.1056/NEJMp2103872

10. Ehrenstein V, Kharrazi H, Lehmann H, Taylor CO. Obtaining Data From Electronic Health Records. Tools and Technologies for Registry Interoperability, Registries for Evaluating Patient Outcomes: A User's Guide, 3rd Edition, Addendum 2 [Internet]. Agency for Healthcare Research and Quality (US); 2019. Available: https://www.ncbi.nlm.nih.gov/books/NBK551878/

11. Butcher CJ, Hussain W. Digital healthcare: the future. Future Healthc J. 2022;9: 113–117. doi:10.7861/fhj.2022-0046

12. Al-Shorbaji N. Improving Healthcare Access through Digital Health: The Use of Information and Communication Technologies. Healthcare Access. IntechOpen; 2021. doi:10.5772/intechopen.99607

13. World Health Organization WH. The World Health Report 2007: A Safer Future : Global Public Health Security in the 21st Century. World Health Organization; 2007.

14. Hub System Improves Health Laboratory Outreach. In: Uganda Radionetwork [Internet]. Mar 2017 [cited 24 Jul 2021]. Available: https://ugandaradionetwork.net/story/hub-system-improves-health-laboratory-outreach

15. Joloba M, Mwangi C, Alexander H, Nadunga D, Bwanga F, Modi N, et al. Strengthening the Tuberculosis Specimen Referral Network in Uganda : The Role of Public-Private Partnerships. 2016;213: 41–46. doi:10.1093/infdis/jiw035

16. Cheng B, Cunningham B, Boeras DI, Mafaune P, Simbi R, Peeling RW. Data connectivity: A critical tool for external quality assessment. Afr J Lab Med. 2016;5: 535. doi:10.4102/ajlm.v5i2.535

17. World Bank. Disease Surveillance, Emergency Preparedness and Response in Eastern and Southern Africa - World | ReliefWeb. 18 Mar 2021 [cited 26 May 2023]. Available: https://reliefweb.int/report/world/disease-surveillance-emergency-preparedness-and-response-eastern-and-southern-africa

18. World Health Organisation. WHO guideline Recommendations on Digital Interventions for Health System Strengthening. WHO guideline Recommendations on Digital Interventions for Health

System Strengthening. World Health Organization; 2019. Available: https://www.ncbi.nlm.nih.gov/books/NBK541888/

19. Digital Square. Supporting the next generation of digital health leaders in Africa. In: Digital Square [Internet]. 6 Oct 2022 [cited 6 Dec 2022]. Available: https://digitalsquare.org/blog/2022/10/6/supporting-the-next-generation-of-digital-health-leaders-in-africa

20. Jain S, Nehra M, Kumar R, Dilbaghi N, Hu TonyY, Kumar S, et al. Internet of medical things (IoMT)-integrated biosensors for point-of-care testing of infectious diseases. Biosens Bioelectron. 2021;179: 113074. doi:10.1016/j.bios.2021.113074

21. Truenat TB Test - Diagnosis, resistance testing, COVID-19 - TBFacts. [cited 4 Jul 2021]. Available: https://tbfacts.org/truenat/

22. World Health Organization. Improving the diagnosis and treatment of smear-negative pulmonary and extrapulmonary tuberculosis among adults and adolescents : recommendations for HIV-prevalent and resource-constrained settings. World Health Organization; 2007. Report No.: WHO/HTM/TB/2007.379. Available: https://apps.who.int/iris/handle/10665/69463

23. Kohli R, Tan SS-L. Electronic Health Records: How Can IS Researchers Contribute to Transforming Healthcare? MIS Q. 2016;40: 553–574.

24. Labrique AB, Wadhwani C, Williams KA, Lamptey P, Hesp C, Luk R, et al. Best practices in scaling digital health in low and middle income countries. Glob Health. 2018;14: 103. doi:10.1186/s12992-018-0424-z

25. Nsoesie EO, Oladeji O, Sengeh MD. Digital platforms and non-communicable diseases in sub-Saharan Africa. Lancet Digit Health. 2020;2: e158–e159. doi:10.1016/S2589-7500(20)30028-5

26. Gibbons CL, Mangen M-JJ, Plass D, Havelaar AH, Brooke RJ, Kramarz P, et al. Measuring underreporting and under-ascertainment in infectious disease datasets: a comparison of methods. BMC Public Health. 2014;14: 147. doi:10.1186/1471-2458-14-147

27. In Action - DHIS2. [cited 27 Sep 2022]. Available: https://dhis2.org/in-action/

28. Behumbiize P. Uganda DHIS2 Case Based Surveillance System: Would Have Detected the 2016 Yellow Fever Outbreak in Uganda. TEXILA Int J Acad Res. 2017;4: 164–170. doi:10.21522/TIJAR.2014.04.02.Art016

29. Sanga ES, Mukumbang FC, Mushi AK, Lerebo W, Zarowsky C. Understanding factors influencing linkage to HIV care in a rural setting, Mbeya, Tanzania: qualitative findings of a mixed methods study. BMC Public Health. 2019;19: 383. doi:10.1186/s12889-019-6691-7

30. Zawedde-Muyanja S, Katamba A, Cattamanchi A, Castelnuovo B, Manabe YC. Patient and health system factors associated with pretreatment loss to follow up among patients diagnosed with tuberculosis using Xpert® MTB/RIF testing in Uganda. BMC Public Health. 2020;20: 1855. doi:10.1186/s12889-020-09955-0

31. Vanqa N, Hoddinott G, Mbenyana B, Osman M, Meehan S-A. Linkage to TB care: A qualitative study to understand linkage from the patients' perspective in the Western Cape Province, South Africa. PLoS ONE. 2021;16: e0260200. doi:10.1371/journal.pone.0260200

32. Maraba N, Chihota V, McCarthy K, Churchyard GJ, Grant AD. Linkage to care among adults being investigated for tuberculosis in South Africa: pilot study of a case manager intervention. BMJ Open. 2018;8: e021111. doi:10.1136/bmjopen-2017-021111

33. The Republic of Uganda Ministry of Health. National Tuberculosis and Leprosy Control Programme Tuberculosis Specimen Referral System Clinician Handbook. RD 001. 2019.

34. Olu O, Muneene D, Bataringaya JE, Nahimana M-R, Ba H, Turgeon Y, et al. How Can Digital Health Technologies Contribute to Sustainable Attainment of Universal Health Coverage in Africa? A Perspective. Front Public Health. 2019;7. Available: https://www.frontiersin.org/articles/10.3389/fpubh.2019.00341

35. Bogner E, Warszawski E. IP Address and Routing Schemes for Overlay Network. US20230142342A1, 2023. Available: https://patents.google.com/patent/US20230142342A1/en

36. Bhardwaj VK. Computer network switching system. US5274631A, 1993. Available: https://patents.google.com/patent/US5274631A/en

37. Falzon D, Timimi H, Kurosinski P, Migliori GB, Gemert WV, Denkinger C, et al. Digital health for the End TB Strategy : developing priority products and making them work. Eur Respir J. 2016; 29–45. doi:10.1183/13993003.00424-2016

38. Falzon D, Jaramillo E, Weyer K, Joos G, Raviglione M. Digital health to end tuberculosis in the Sustainable Development Goals era : achievements , evidence and future perspectives. Eur Respir J. 2018. doi:10.1183/13993003.01632-2017

39. Defeat TB. [cited 1 Dec 2021]. Available: https://www.urc-chs.com/projects/defeat-tb/

40. Babirye D, Shete PB, Farr K, Nalugwa T, Ojok C, Nantale M, et al. Feasibility of a short message service (SMS) intervention to deliver tuberculosis testing results in peri-urban and rural Uganda. 16: 100110. doi:10.1016/j.jctube.2019.100110

Figure 1

Click here to access/download;Figure;Fig 1.tif

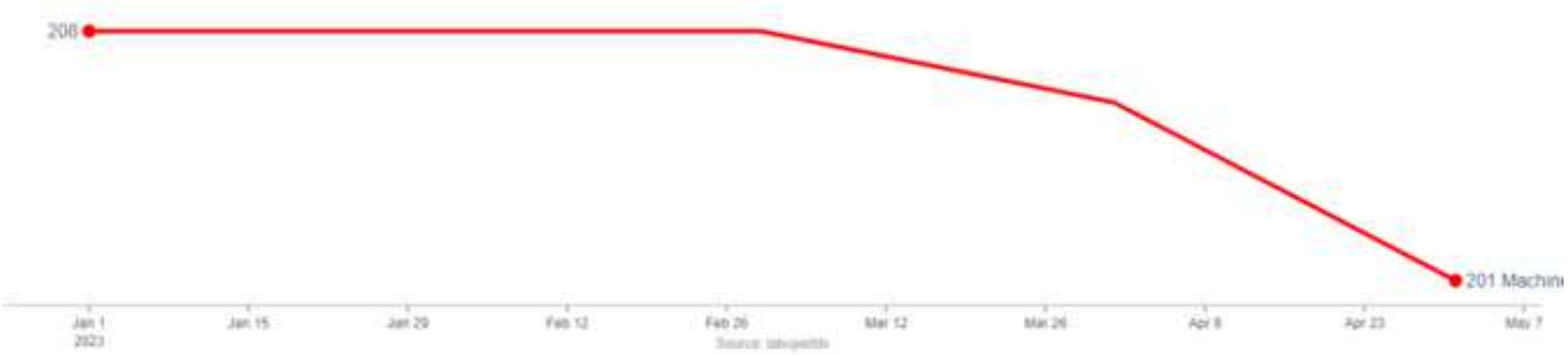

Figure 2

Click here to access/download;Figure;Fig 2.tif

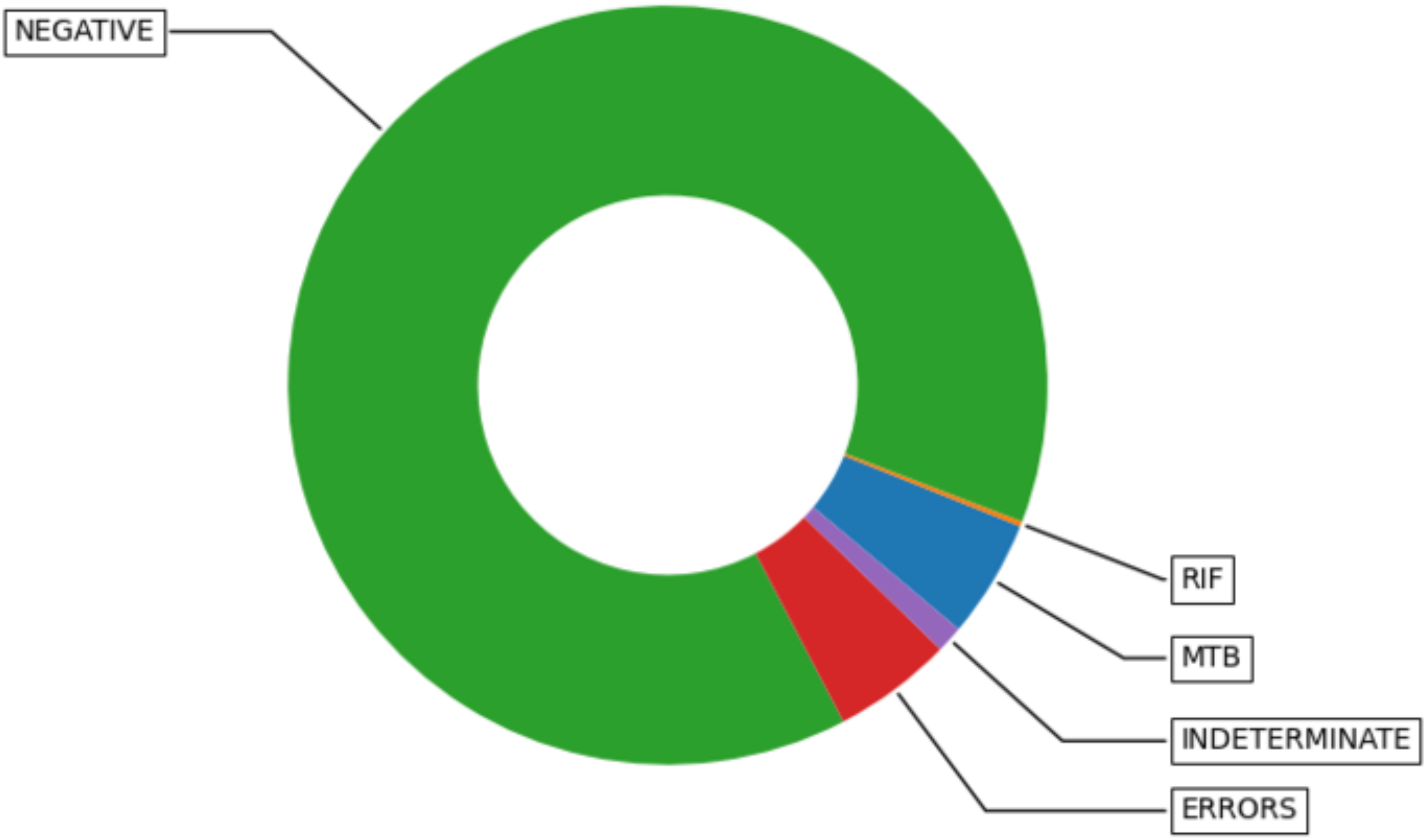

Figure 3

Click here to access/download;Figure;Fig 3.tif

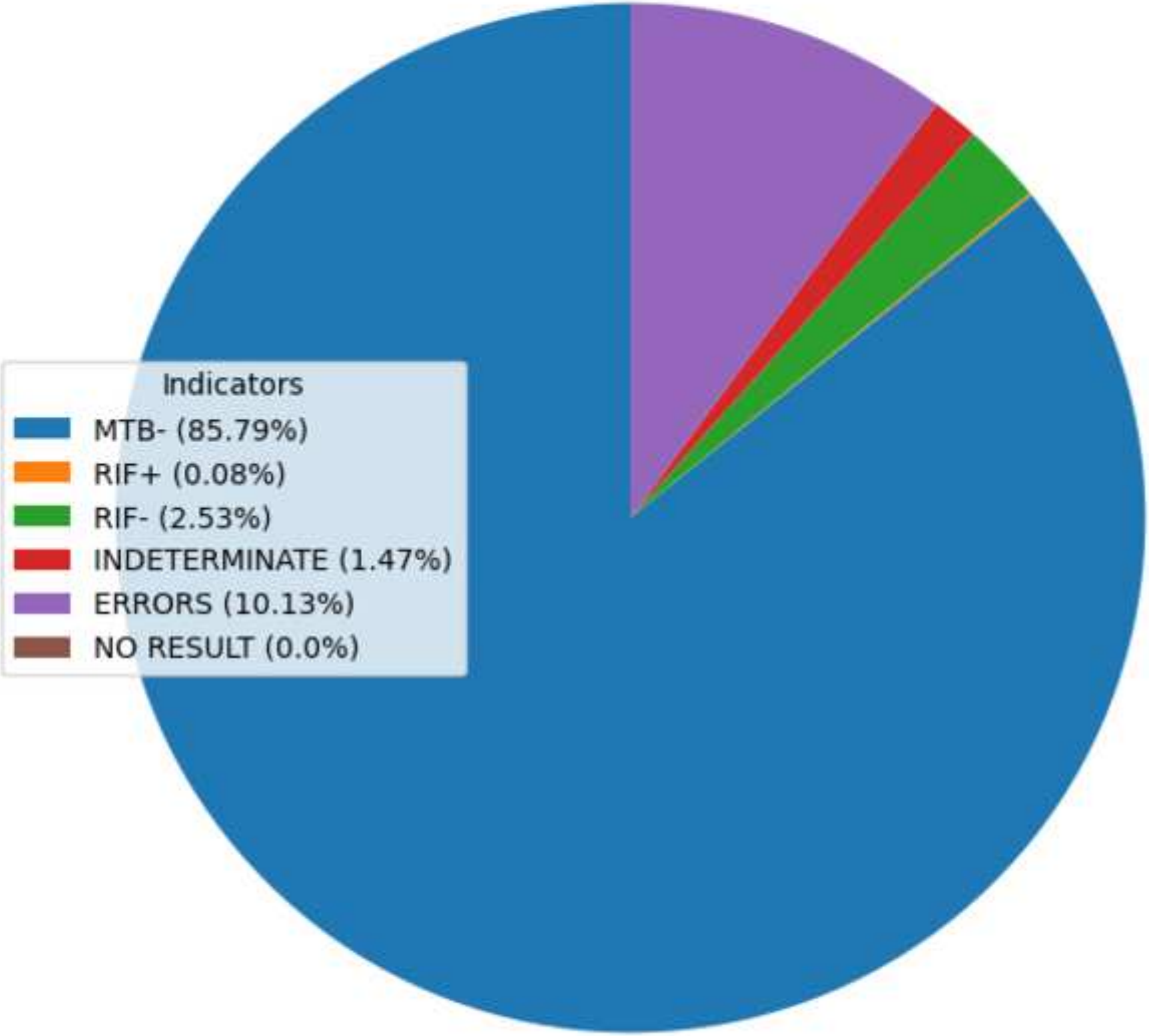

Figure 4

Click here to access/download;Figure;Fig 4.tif

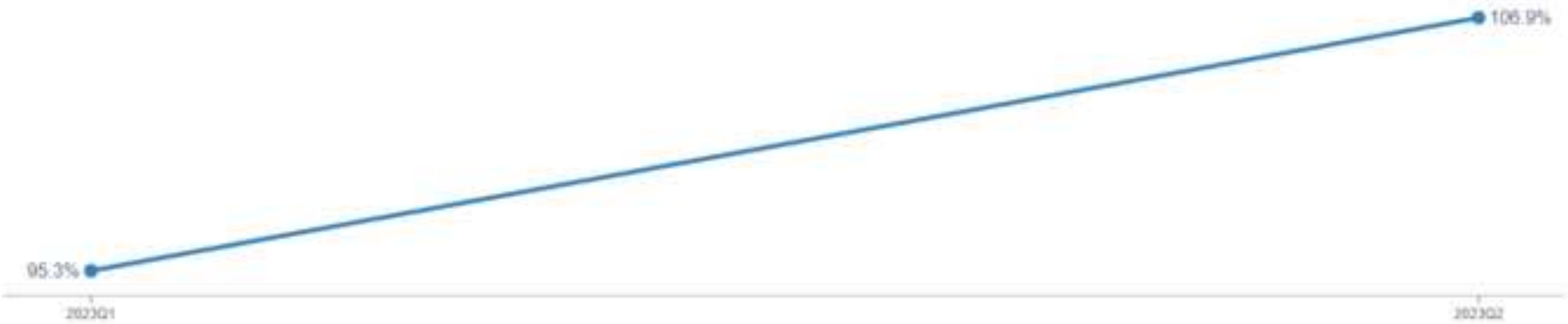

Figure 5

Click here to access/download;Figure;Fig 5.tif

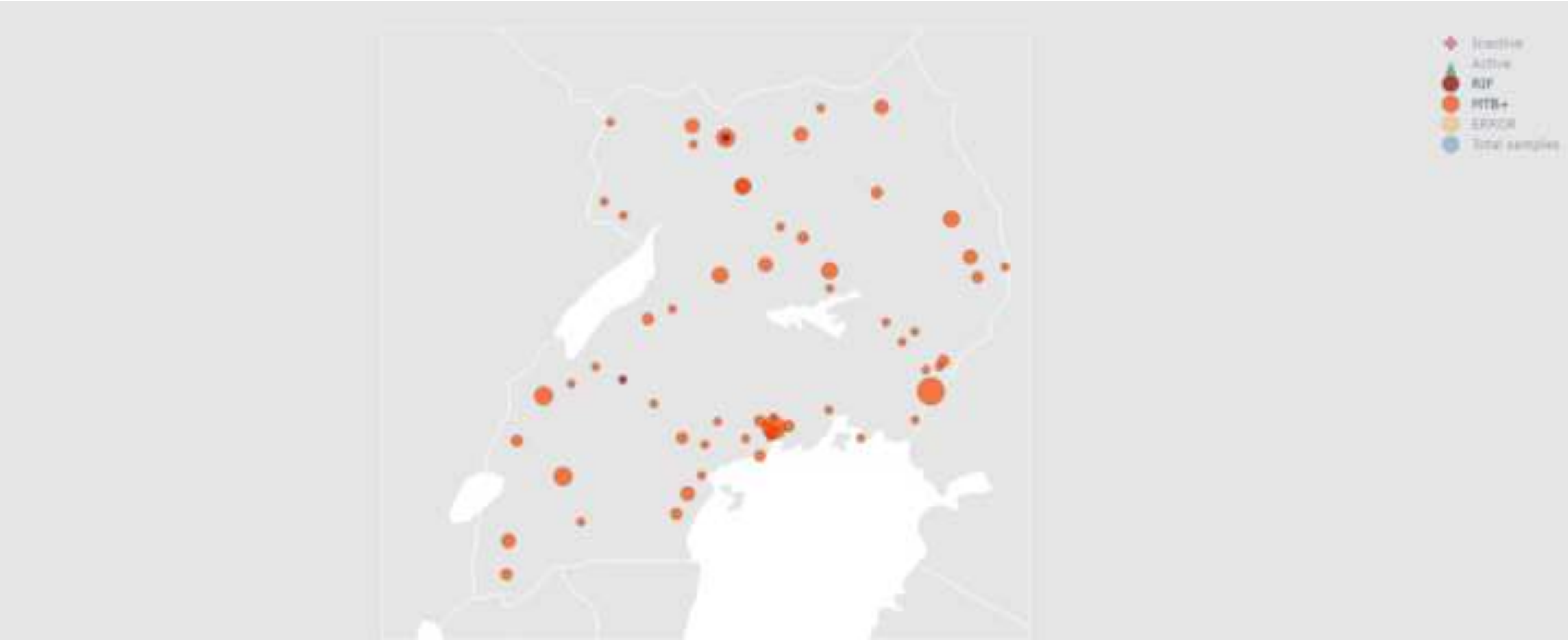

Figure 6

Click here to access/download;Figure;Fig 6.tif

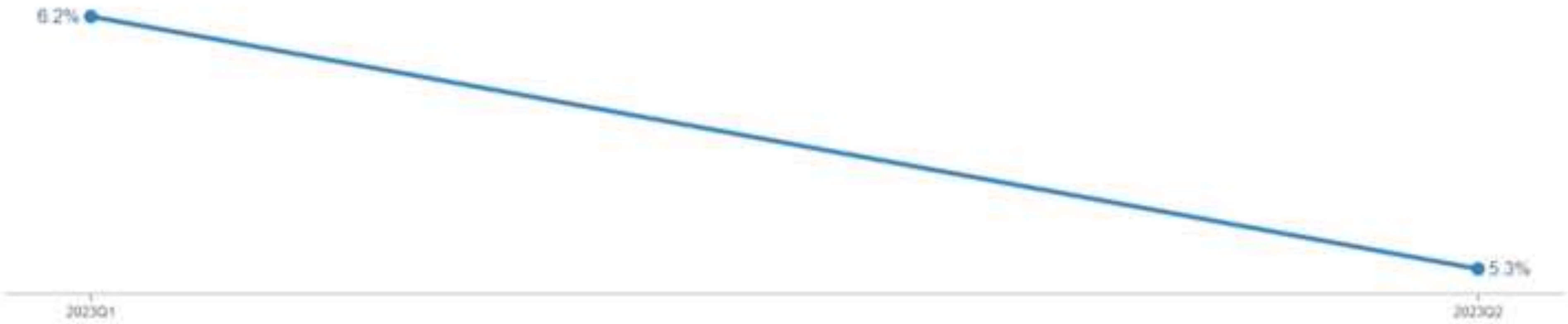

Supplement: Attachment — Submitted filename: PDIG-D-23-00344_reviewer.pdf [file pdig.0000566.s008.pdf]
