# Supplementary material for: Beyond diagnostic connectivity: Leveraging digital health technology for the real-time collection and provision of high-quality actionable data on infectious diseases in Uganda
Source: PLOS Digit Health. 2024 Aug 23;3(8):e0000566. doi: 10.1371/journal.pdig.0000566 (PMC11343378; doi:10.1371/journal.pdig.0000566)
Supplement: Attachment — Submitted filename: Response to Reviewers.docx [file pdig.0000566.s009.docx]

PDIG-D-23-00344
Beyond diagnostic connectivity: leveraging digital health technology for the real-time collection and provision of high-quality actionable data on infectious diseases in Uganda
PLOS Digital Health

Dear editorial office at PLOS Digital Health, I am pleased to submit our revised manuscript with all previous concerns addressed, to the best of our ability.

NB: The Line numbering referenced below is in line with the copy with tracked changes.

Please receive them below in blue font.

Journal Requirements:

1. We do not publish any copyright or trademark symbols that usually accompany proprietary names, eg  ©, ®, ™  (e.g. next to drug or reagent names). Please remove all instances of trademark/copyright symbols throughout the text, including ® on page 22 & ™ on pages 8, 10, 11 & 12.

Response:

The ® on the citation “Zawedde et al.,” (previously page 22, currently page 32) has been removed,

The ™ previously on pages 8, 10, 11, and 12 have been removed from the current 9, 12, 13, and 15 respectively.

2. In the online submission form, you indicated that "The datasets used and/or analyzed during the current study are owned by the government of Uganda and are therefore only available from the corresponding author on reasonable request". All PLOS journals now require all data underlying the findings described in their manuscript to be freely available to other researchers, either 1. In a public repository, 2. Within the manuscript itself, or 3. Uploaded as supplementary information.
This policy applies to all data except where public deposition would breach compliance with the protocol approved by your research ethics board. If your data cannot be made publicly available for ethical or legal reasons (e.g., public availability would compromise patient privacy), please explain your reasons by return email and your exemption request will be escalated to the editor for approval. Your exemption request will be handled independently and will not hold up the peer review process, but will need to be resolved should your manuscript be accepted for publication. One of the Editorial team will then be in touch if there are any issues.

Additional Editor Comments (if provided):
Response: We have added the data as supplementary files as excel sheets for the data collected between; 01^st^ May 2022 and 31^st^ May 2023. A section has been added to Line 788 for this section.

We recommend that the statistical model employed in the methodology and data analysis sections be clearly stated in the manuscript. The data should be available to the public as a supplementary file for scientific proof.

Response: Lines 524-579. A section has been added to the methodology, showing the full description of the statistical model employed to get the visualized dashboards.

[Note: HTML markup is below. Please do not edit.]

Reviewers' comments:

Reviewer's Responses to Questions

<font color="black"><b>Comments to the Author</b><br><br>
1. Does this manuscript meet PLOS Digital Health’s <a href="<https://journals.plos.org/digitalhealth/s/journal-information#loc-criteria-for-publication>" target="_blank">publication criteria</a>? Is the manuscript technically sound, and do the data support the conclusions? The manuscript must describe methodologically and ethically rigorous research with conclusions that are appropriately drawn based on the data presented.</font>


Reviewer #1: Yes

Reviewer #2: Yes

Reviewer #3: Partly

Reviewer #4: No

--------------------

<font color="black">2. Has the statistical analysis been performed appropriately and rigorously?</font>

Reviewer #1: N/A

Reviewer #2: No

Reviewer #3: N/A

Reviewer #4: No

--------------------

<font color = "black">3. Have the authors made all data underlying the findings in their manuscript fully available (please refer to the Data Availability Statement at the start of the manuscript PDF file)?<br><br> The <a href="<https://journals.plos.org/digitalhealth/s/data-availability>" target="_blank">PLOS Data policy</a> requires authors to make all data underlying the findings described in their manuscript fully available without restriction, with rare exception. The data should be provided as part of the manuscript or its supporting information, or deposited to a public repository. For example, in addition to summary statistics, the data points behind means, medians and variance measures should be available. If there are restrictions on publicly sharing data—e.g. participant privacy or use of data from a third party—those must be specified.</font>

Reviewer #1: No

Reviewer #2: Yes

Reviewer #3: No

Reviewer #4: No

--------------------

<font color = "black">4. Is the manuscript presented in an intelligible fashion and written in standard English?<br/><br/>PLOS Digital Health does not copyedit accepted manuscripts, so the language in submitted articles must be clear, correct, and unambiguous. Any typographical or grammatical errors should be corrected at revision, so please note any specific errors here.</font>

Reviewer #1: Yes

Reviewer #2: Yes

Reviewer #3: Yes

Reviewer #4: Yes

--------------------

<font color = "black">5. Review Comments to the Author<br/><br/>Please use the space provided to explain your answers to the questions above. You may also include additional comments for the author, including concerns about dual publication, research ethics, or publication ethics. (Please upload your review as an attachment if it exceeds 20,000 characters)</font>

Reviewer #1: 1. The paper has a significant solution to a major public concern which will solve a number of issues related to data loses.
2. There is a need to improve the overall paper in regards to language use, especially punctuations and avoiding use of very long sentences.
Response: We have worked on punctuations and rephrasing to align the language, while avoiding long sentences.
3. Arrangement of the overall paper is bad. Rearrange the whole paper to have a logical flow of ideas. Use PlosDigital manuscript format.

Response: We followed the PLOS Digital Health Format, this remains unchanged from the first submitted version, except the addition of “supporting information captions” section added on Line 788.

The current format is therefore as follows;

- Title
- Authors
- Affiliations
- Abstract
- Author Summary
- Introduction
- Results
- Discussion
- Materials and Methods
- Acknowledgments
- References
- Supporting information captions [***Line 788***]

We have only rearranged the flow within the methodology to represent the logical flow of the project, where we placed the ***Training of users and other relevant human resources (****Lines 490-495****)*** just before the pilot phase.

Results
The results section is poorly written

Response: We have reviewed and improved the wording within the results presentation/flow to make the section as comprehendible as possible to the readers.

The diagrams and the charts need to be high quality and more clear
Response: We have used PACE to get the best quality of diagrams/charts for the manuscript.

4. The discussion needs improvement on the use of the machines and how it benefits the patients directly
Response Lines 210-212: We have weaved this into the existing discussion in each section; firstly, with an introduction to the section, showing how the implementation of diagnostic connectivity would potentially benefit patients directly by facilitating faster diagnoses, by enabling quicker treatment initiation and more effective management of patients through disease control programs.

1. Lines 267-269. **Diagnostic network connectivity**: After discussing the potential of increasing real-time data collection from various sites, we have added emphasis on how the immediate transmission of data from machines to a central database allows for quicker diagnosis and, subsequently, faster initiation of appropriate treatments for patients.
2. Lines 302-311 **Utilization of actionable data**: We have added a description of how the utilization of actionable data from the diagnostic machines helps improve patient management through various stakeholder avenues.

For example, stock and machine utilization metrics prompt health workers to ensure stock availability (for uninterrupted service delivery) and faster diagnosis for patients, which can lead to quicker initiation of appropriate treatments. This helps patients receive the right treatment faster in the case of tuberculosis disease, resulting in improved health outcomes and potentially reducing the spread of the disease. This serves as an example for other disease control programs to bench mark from.

Additionally, the near real-time hotspot mapping provides actionable surveillance data that leads to prompt public health action through community out-reaches to underserved areas, eventually protecting at-risk populations.

3. Lines 332-335 **Building technical capacity**: We have discussed how building human resource capacity contributes to better patient outcomes since training personnel how to use the diagnostic connectivity solution to troubleshoot issues enables them to provide uninterrupted service delivery, leading to faster patient diagnoses and treatments.
4. Lines 393-396 **Linkage to care**: We have added points about how the connectivity solution improves patient care. For example, how timely linkage to care can be directly related to a faster initiation of anti-TB therapy, which benefits patients by reducing their symptoms and the duration of their illness. We have also discussed how the prompt transmission of critical results to clinicians helps them make timely decisions about patients' treatment plans.

We hope this is satisfactory.

5. The readers would benefit to see improvement and  benefits of digitalizing the diagnostic machines by showing a chart of how much loses of data were there before and how much has been saved.

Response Lines 214-225: We have not been able to put a chart, but have addressed this very important point from the reviewer with the following data and contextualization in the discussion section under “**Diagnostic network connectivity**”;

Prior to the implementation of diagnostic connectivity, data loss was a significant challenge in the diagnostic network of Uganda. Inconsistent data handling and manual record-keeping led to lost or misreported results, delaying patient care and impacting surveillance efforts. For instance, in 2020, the NTLP received notifications for 60,887 TB cases, representing 68% of the expected total cases [19]. This indicates that around 29,000 TB cases either went undiagnosed or were diagnosed but not reported to the NTLP. In 2020, only one-third of the estimated 1,500 drug-resistant TB (DR-TB) cases were diagnosed and reported to the NTLP, while in 2021, 615 of the anticipated 1,424 RR/MDR-TB patients were detected across GeneXpert and Truenat sites nationwide, resulting in an annual case detection rate (CDR) of 43.2% [20]. This indicates the many TB cases that were left unrecorded due to paper-based errors or delays. However, automated data transmission and real-time data access can nearly eliminate such data losses.

6. As far as the background states on how big the problem was before, kindly explain the problems faced by the patients and how this strategy is going to help patients.
Response Lines 109-116: We have added this to the background (introduction) as follows;

Patient samples can now be digitally tracked, unlike in the recent past. Additionally, the follow-up data is electronically retrievable in order to bridge the gap between data discrepancies that are frequently encountered with the transfer-ins and outs as patients move from one area of diagnosis, referral, or treatment to another in search of health services. Furthermore, the patients who complete treatment are not well documented for cure rate computation, making it difficult to have accurate treatment success rates to inform treatment strategy efforts. In the diagnostic connectivity solution, the electronic documentation for cure/failed after anti-TB treatment completion is highly prioritized to facilitate accurate accountability that eases anti-TB treatment program evaluation.

Top of Form

Reviewer #2: An important manuscript discussing Uganda's efforts to utilize digital technology for the diagnosis, surveillance, and patient care of infectious diseases.
Here are some suggestions for further clarification or questions you might consider:
1.      Specify Statistical Methodology:
Request more details on the statistical methodology employed in the study. Knowing the specific methods used can help understand the reliability of the findings and the significance of any reported differences.

Response: Addressed as highlighted elsewhere; Lines 524-579.

2.      Error Rates for Each Technology:
Highlight the importance of knowing the error rates for each digital technology used, including GeneXpert, Truenat, and CAD4TB. It would be helpful to understand the nature of these errors and if they have any clinical implications.

Response Lines 336-361: We have incorporated a new section in the discussion, to discuss the importance of the diagnostic machine errors and their clinical implications as follows;

**Equipment performance monitoring and reporting**

The error rates for each digital technology used, such as GeneXpert, Truenat, and CAD4TB, play a critical role in ensuring the reliability and clinical safety of these diagnostic systems. Monitoring these error rates provides insights into the nature and frequency of errors, which may have direct implications for patient care and outcomes.

For “GeneXpert”, error rates can stem from various factors such as sample quality, system malfunctions, or operator errors. Understanding the nature of these errors allows for targeted interventions, including retraining equipment operators, air conditioning laboratories in arid areas, calibrating diagnostic equipment, or enhancing sample processing protocols. As shown in Figure 6, monitoring trends in “GeneXpert” error rates enables proactive maintenance and system optimization from the connectivity solution embedded prompts. This initiative can enhance diagnostic accuracy and ensure timely treatment initiation for patients.

Similarly, for Truenat, error rates could arise from issues such as reagent quality, equipment calibration, or procedural mistakes. Analyzing these errors helps users to identify areas for improvement in testing protocols and equipment maintenance. This ensures that the “Truenat” machines remain a reliable and efficient diagnostic tool for detecting TB and other diseases.

CAD4TB, a digital chest X-ray system, can also experience errors related to image quality, interpretation challenges, or algorithm performance. Although we did not collect any errors during our period of interest, understanding the sources of such errors when they occur would be crucial for minimizing false positive and negative likelihood scores, thereby improving the overall accuracy of TB risk stratification and diagnosis.

Therefore, healthcare providers can ensure that these diagnostic tools operate at optimal performance levels thereby minimizing clinical implications such as delayed or incorrect diagnoses for patients through the continuous monitoring and analyzing error rates for these digital technologies. This would lead to better patient outcomes and more effective disease management by disease control programs.

3.      Comparison of Technologies:
While the main objective may not be a direct comparison of the systems, it would be valuable to know if there were any statistically significant differences in error rates between GeneXpert and Truenat. This information can provide insights into the effectiveness of each technology.

Response: The primary focus of our study was not to directly compare the performance of GeneXpert and Truenat in terms of error rates. Our study methodology was designed to share the potential of diagnostic connectivity-beyond data collection for the real-time collection and provision of high quality actionable data on infectious diseases in Uganda rather than evaluating diagnostic comparisons. As such, the data did not account for potential biases that might arise from a direct comparison of the two technologies.

While insights into potential differences in error rates between GeneXpert and Truenat would be valuable, our current study does not provide statistically significant comparisons. Any observations regarding differences in error rates between the two systems should be interpreted with caution, as the study design and data collection approach did not control for factors that might influence these comparisons.

Future studies with a specific focus on comparing the error rates and diagnostic effectiveness of GeneXpert and Truenat could provide more robust insights into the relative performance of these technologies.

We have therefore listed this as a limitation, Lines 420-422 as follows and added the future research clause in the conclusion;

“Moreover, our study did not aim to directly compare error rates between GeneXpert and Truenat as molecular diagnostics. Therefore, any potential differences observed should be interpreted with caution, as potential biases were not controlled for."

4.      Nature of Errors:
Inquire about the nature of errors, whether they were related to diagnosis, drug resistance detection, or any other aspect. Understanding the type of errors can help gauge the practical implications and areas for improvement.

Response: Understanding the nature of errors in diagnostic technologies is crucial for assessing their practical implications and identifying areas for improvement. Errors can arise from various aspects, including diagnosis, drug resistance detection, or technical issues. In this implementation, we collected errors that were machine function related (technical), and not diagnostic accuracy (false positive/negative) related. We have therefore addressed this by adding this to the discussion of errors Lines 362-372:

**Practical implications of errors and areas for improvement**

Understanding the nature of errors in diagnostic technologies is crucial for assessing their practical implications and identifying areas for improvement. In our implementation, we focused on machine function-related errors, rather than diagnostic accuracy issues such as false positives or false negatives. Technical problems may arise from sample quality, equipment malfunctions, or operator errors, resulting in the need for reruns and causing potential delays in diagnosis and treatment. These delays could lead to significant challenges for patients, such as out-of-pocket transportation costs for repeated visits to testing facilities or the risk of pre-treatment loss to follow-up.

In Uganda, these challenges are particularly concerning due to missed TB screening opportunities (Wamulima et al., 2022) and delays in diagnosis and treatment for vulnerable populations facing healthcare navigation challenges (DAHW, 2023). The action-driven dashboard, enhanced by diagnostic connectivity, helps users quickly identify problem areas and take targeted action to improve patient outcomes.

Top of Form

5.      CAD4TB Data:
Express the need for information on the error rates associated with CAD4TB. This would contribute to a comprehensive understanding of the performance of all three digital technologies.

Response: While the error rates associated with CAD4TB would contribute to a comprehensive understanding of the performance of all three digital technologies, we did not collect this data from the digital chest X-ray during the study period. This was due to limitations in data availability, and technical constraints at the time of the study. As a result, we are unable to provide insights into the error rates for CAD4TB at this time.

We have included this in the limitations section as follows on Lines 417-419;

“While understanding error rates associated with CAD4TB would offer a more comprehensive view of the performance of all three digital technologies, we were unable to collect this data from the digital chest X-ray during the study period due to technical constraints to collect this data.”

6.      Impact of Errors:
Consider discussing the potential impact of errors on patient outcomes and public health. This could add depth to the analysis of the study's implications.

Response: We have addressed this in number 4 above; please refer to Lines 362-372 in the manuscript

7.      Suggestions for Future Research:
Propose that future research might focus on a more direct comparison of these digital technologies, taking into account error rates and specific functionalities.

Responses on Line 433-437: Future research should focus on a direct comparison of diagnostics through diagnostic connectivity, considering error rates and complementary testing. This could provide valuable insights into their relative effectiveness in terms of time and money, enabling more informed decisions in the selection and deployment of diagnostic tools to enhance healthcare delivery and disease control.

Line 39 in the abstract has also been edited to add this context with the words, “including error analysis”.

In conclusion, your observations highlight the importance of clarity and completeness in research reporting. Requesting additional information on statistical methods and specific data related to error rates can enhance the overall understanding of the study's findings and their implications for the use of digital technologies in LMICs.

Response: This is well appreciated. We hope that our revision now fully addresses the raised concerns, while maintaining the completeness of the previous writing.

Reviewer #3: The statistical model employed in the data analysis and the methodology part are not clearly stated in the manuscript; the data should be available to the public as a supplementary file for scientific proof.
Response: This has been addressed; Lines 524-579.

Reviewer #4: This paper needs to elaborate more on the depth of the research. The paper rarely conducted any statistical analysis, although it claimed 'a sophisticated and automated data analysis,  nothing was presented in the paper apart from the pure descriptive statistics. On the qualitative discussion, it was mainly on the description of the connection of devices, without much discussion on its implication for the policy, the community, etc. If the authors hope to establish this as a case study, then we are not seeing how could this be elevated to form a framework of engagement for communities facing similar challenges.
Response: This has been addressed; Lines 524-579.
--------------------

I look forward to corresponding with you in this regard.

Thank you

Sincerely,

Dennis Mujuni

Lead Author, Corresponding Author
